# Lipid vesicles containing transferrin receptor binding peptide TfR-T_12_ and octa-arginine conjugate stearyl-R_8_ efficiently treat brain glioma along with glioma stem cells

**DOI:** 10.1038/s41598-017-03805-7

**Published:** 2017-06-14

**Authors:** Li-Min Mu, Ying-Zi Bu, Lei Liu, Hong-Jun Xie, Rui-Jun Ju, Jia-Shuan Wu, Fan Zeng, Yao Zhao, Jing-Ying Zhang, Wan-Liang Lu

**Affiliations:** 1 Beijing Key Laboratory of Molecular Pharmaceutics and New Drug System, State Key Laboratory of Natural and Biomimetic Drugs, and School of Pharmaceutical Sciences, Peking University, Beijing, China; 2 Department of Pharmaceutical Engineering, Beijing Institute of Petrochemical Technology, Beijing, China

## Abstract

Surgery and radiotherapy cannot fully remove brain glioma; thus, chemotherapy continues to play an important role in treatment of this illness. However, because of the restriction of the blood-brain barrier (BBB) and the regeneration of glioma stem cells, post-chemotherapy relapse usually occurs. Here, we report a potential solution to these issues that involves a type of novel multifunctional vinblastine liposomes equipped with transferrin receptor binding peptide TfR-T_12_ and octa-arginine conjugate stearyl-R_8_. Studies were performed on brain glioma and glioma stem cells *in vitro* and were verified in brain glioma-bearing mice. The liposomes were transported across the BBB, killing brain glioma and glioma stem cells via the induction of necrosis, apoptosis and autophagy. Furthermore, we reveal the molecular mechanisms for treating brain glioma and glioma stem cells via functionalized drug lipid vesicles.

## Introduction

Brain glioma is the most frequent primary malignant brain tumor, and the mean survival time of patients with this illness is only one year^1, 2^. Neither surgery nor radiotherapy alone can cure brain glioma. Therefore, chemotherapy has typically been incorporated into therapeutic approaches that combine surgical resection and radiotherapy to eliminate cancer cells. However, chemotherapy is often an ineffective treatment for brain glioma because of the existence of the blood-brain barrier (BBB). The BBB, which consists of vascular endothelial cells in the central nervous system, restricts the transport of substances and drugs into the brain^3^, leading to the failure of chemotherapy. Furthermore, the ineffectiveness of chemotherapy arises from a small population of glioma stem cells (GSCs) amidst brain glioma cells^4^. Glioma stem cells have been regarded as the initiating cells, which have a high tumorigenic potential but a low proliferation rate. These cells express high levels of ATP-binding cassette transporters (ABC transporters) and possess the capacity for self-renewal, resulting in refractoriness and relapse of brain glioma^5^. Therefore, how to transport of drug to across the BBB and how to eliminate brain glioma cells and glioma stem cells remain important scientific issues. In this study, we propose a type of functionalized lipid vesicles, named as multifunctional vinblastine liposomes, which potentially transfer vinblastine across the BBB and subsequently eliminate brain glioma cells and glioma stem cells when equipped with the transferrin receptor binding peptide TfR-T_12_ and octa-arginine conjugate stearyl-R_8_.

Unique pathways exist to transport normal physiological substances across the BBB, including receptor-mediated transcytosis (RMT) and adsorptive-mediated transcytosis (AMT)^6^. Recent evidence has shown that transferrin receptors (TfRs) are highly expressed in brain capillary endothelial cells and are responsible for the uptake of transferrin by RMT^7^. Moreover, electric charges are involved in AMT-mediated transcytosis, and positively charged physiological substances can be transferred across the BBB through AMT by interacting with the negative charges on the surface of brain capillary endothelial cells^8^. In addition, TfRs are overexpressed on brain glioma cells and glioma stem cells; these cells are rich in negative charges because of their increased activity compared to that of normal cells^9^. Accordingly, these features can be used both to facilitate the transcytosis of functionalized drug carriers across the BBB and to potentiate the endocytosis of the carriers by brain glioma cells and glioma stem cells.

Liposomes are nanoscale lipid vesicles consisting of a phospholipid bilayer with an aqueous interior core^10^. Therefore, they can be used to load both hydrophobic and hydrophilic drug molecules. Previous preclinical and clinical investigations have proven that nanosized drug liposomes show improved accumulation in tumor tissues because of the enhanced permeability and retention (EPR) effect, resulting in a superior therapeutic effect whereas a reduced systemic side effect^11^. In this study, multifunctional vinblastine liposomes were fabricated to achieve these goals.

As a model drug carried by the vesicles, vinblastine is a vinca alkaloid extracted from *Catharanthus roseus* ^12^. It is an effective treatment for various cancers and is thus listed on the World Health Organization’s (WHO) model list of essential medicines^13^. Vinblastine is capable of binding β-tubulin and disrupting the function of microtubules during mitosis, thereby killing tumor cells^14^. Although vinblastine is also effective in killing brain glioma cells *in vitro*, it is ineffective in clinical chemotherapy because of the restriction of the BBB. As an anticancer drug, vinblastine is incorporated into liposomes.

To endow the vesicles with specific functions, proper functional materials should be selected. Transferrin receptor binding peptide TfR-T_12_, which consists of 12 amino acids (THRPPMWSPVWP), is a synthetic peptide obtained by phage display, and is able to bind a different site on TfRs compared with transferrin^15, 16^. In this study, TfR-T_12_ was chemically conjugated with a pegylated lipid derivative, 3-(N-succinimidyloxyglutaryl)aminopropyl- polyethyleneglycol(2000)- carbamyl distearoyl phosphatidylethanolamine (NHS-PEG_2000_-DSPE), and used as a functional material to construct the multifunctional lipid vesicles. Stearyl-R_8_ is a conjugate of an octa-arginine (RRRRRRRR) with a stearyl group. Because of its multi-cationic centers, stearyl-R_8_ was also used as a functional material to transfer lipid vesicles across the BBB and mediate the endocytosis of the lipid vesicles by brain glioma cells and glioma stem cells^17, 18^.

Here, we show that the constructed multifunctional vinblastine liposomes lead to transcytosis across the BBB and endocytosis into brain glioma cells and glioma stem cells, resulting in necrosis, apoptosis or autophagy and the elimination of both brain glioma cells and glioma stem cells. The mechanisms of necrosis, apoptosis and autophagy were further revealed at the organelle and molecular levels. The results evidenced the efficacy of multifunctional vinblastine liposomes in the treatment of brain glioma and unveiled their potential as a new chemotherapy strategy for brain glioma and glioma stem cells.

## Results

### Synthesis of TfR-T_12_-PEG_2000_-DSPE and characterization of the liposomes

**Figure 1:**
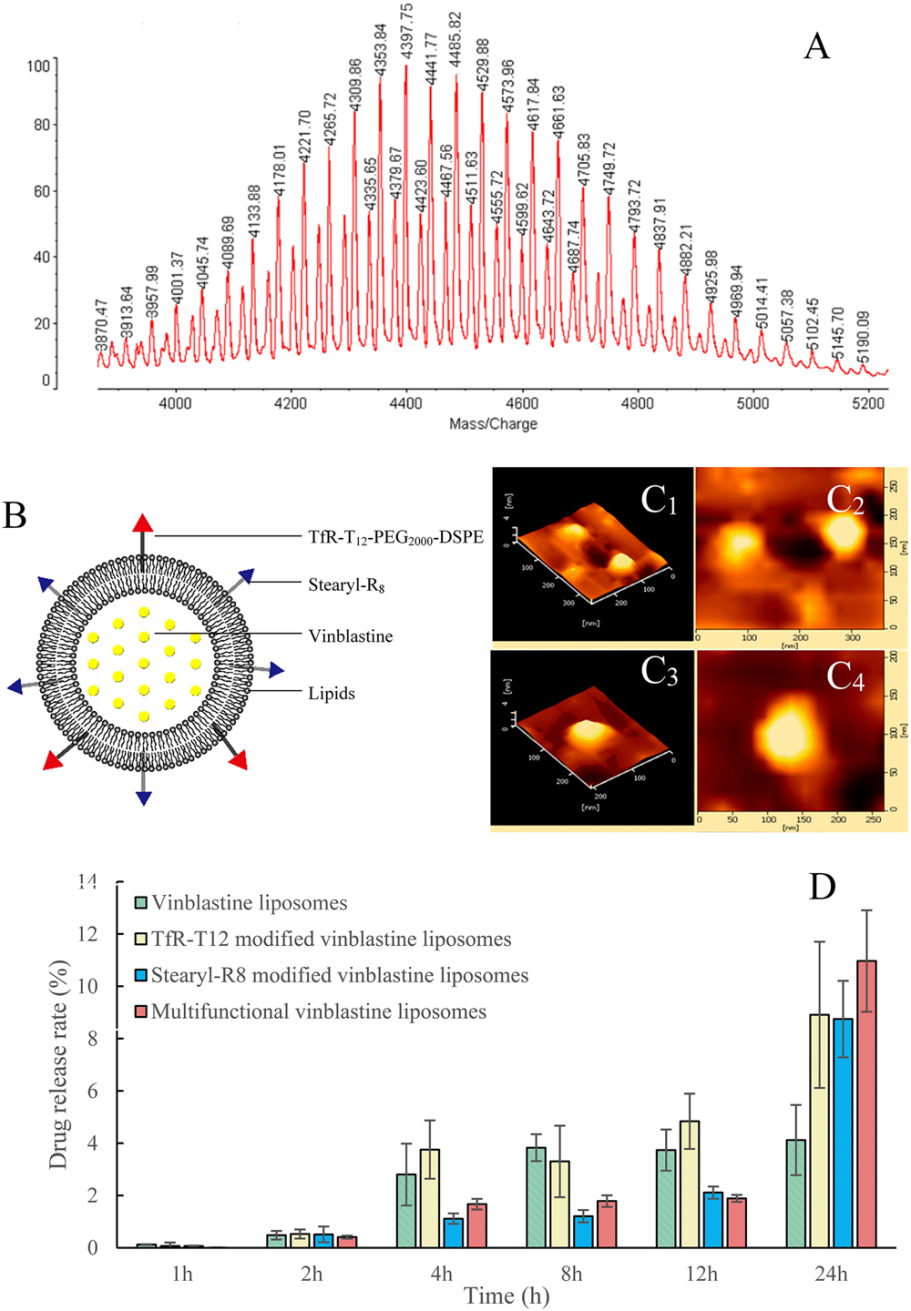
Characterization of functional material TfR-T_12_-PEG_2000_-DSPE and multifunctional vinblastine liposomes. (**A**) MALDI-TOF-MS spectrum of TfR-T_12_-PEG_2000_-DSPE. (**B**) Schematic representation of multifunctional vinblastine liposomes. (**C**) AFM images: 3-dimensional (**C**_**1**_) and 2-dimensional (**C**_**2**_) images of vinblastine liposomes; 3-dimensional (**C**_**3**_) and 2-dimensional (**C**_**4**_) images of multifunctional vinblastine liposomes. (**D**) Release rates of vinblastine in blood components containing phosphate buffered solution. The data are presented as the means ± standard deviation (n = 3).

The matrix-assisted laser desorption/ionization time-of-flight mass spectrometry (MALDI-TOF-MS) spectrum (Fig. 1A) showed that the average mass of the product was m/z 4441.77. The difference of the average mass between the product and PEG_2000_-DSPE (m/z 3000) was equal to the mass of TfR-T_12_ (1490.8), indicating the successful synthesis of TfR-T_12_-PEG_2000_-DSPE.

The structure of the multifunctional vinblastine liposomes is illustrated in the schematic drawing (Fig. 1B). Vinblastine was encapsulated into the inner vesicle of the liposomes using a transmembrane ammonium gradient method^19^. The PEG_2000_-DSPE component of TfR-T_12_-PEG_2000_-DSPE and the stearyl component of stearyl-R_8_ were inserted into the lipophilic bilayer; the TfR-T_12_ and R_8_ components were left on the liposome exterior as the functional groups. Atomic force microscopy (AFM) images indicated that the vinblastine liposomes (Fig. 1C_1,2_) and multifunctional vinblastine liposomes (Fig. 1C_3,4_) were round with smooth surfaces. The drug release rates in the first 24 h were all below 12% at an oscillating rate of 100 revolutions per minute (rpm) in the phosphate buffered saline containing blood components (Fig. 1D).

The encapsulation efficiency, size, polydispersity index (PDI) and zeta potential were characterized (see Supplementary Table S1). The encapsulation efficiency of multifunctional vinblastine liposomes was greater than 96%, and the average size was approximately 110 nm with a narrow polydispersity index (<0.25). The zeta potential was approximately −0.97 ± 0.17 mV.

### Identification of phenotypes

**Figure 2:**
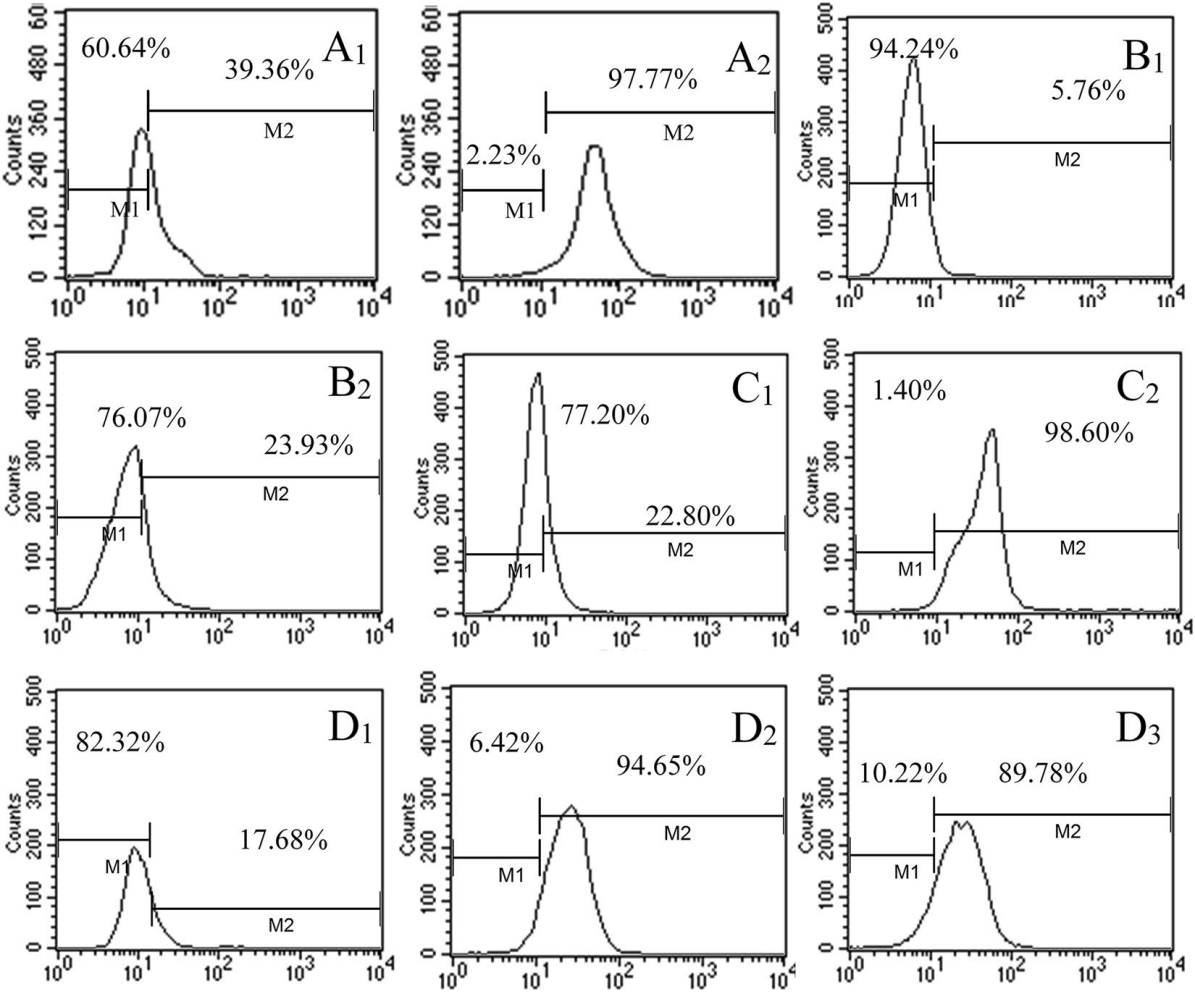
Expression of biomarkers in brain microvascular endothelial cells, brain glioma and glioma stem cells. (**A**) Expression of nestin in brain glioma (**A**_**1**_) and glioma stem cells (**A**_**2**_). (**B**) Expression of ABCG2 transporter in brain glioma (**B**_**1**_) and glioma stem cells (**B**_**2**_). (**C**) Expression of CXCR4 receptor in brain glioma (**C**_**1**_) and glioma stem cells (**C**_**2**_). (**D**) Expression of transferrin receptors in brain microvascular endothelial cells (**D**_**1**_), brain glioma (**D**_**2**_) and glioma stem cells (D_3_).

The expression of ATP-binding cassette subfamily G member 2 (ABCG2), chemokine receptor type 4 (CXCR4) and nestin were identified on human brain glioma cells and glioma stem cells (GSCs). The expression ratios of ABCG2 were 5.76% on brain glioma cells (Fig. 2A_1_) and 23.93% on glioma stem cells (Fig. 2A_2_); those of CXCR4 were 22.80% on brain glioma cells (Fig. 2B_1_) and 98.60% on glioma stem cells (Fig. 2B_2_); those of nestin were 39.36% on brain glioma cells (Fig. 2C_1_) and 97.77% on glioma stem cells (Fig. 2C_2_). The results demonstrated that the expression ratios of ABCG2, CXCR4 and nestin were higher on glioma stem cells than on brain glioma cells.

The expression ratios of TfRs were 17.68% on murine brain microvascular endothelial cells (BMVECs) (Fig. 2D_1_), 94.65% on brain glioma cells (Fig. 2D_2_), and 89.78% on glioma stem cells (Fig. 2D_3_). The results indicated that BMVECs, brain glioma cells and glioma stem cells all expressed TfRs, but the BMVECs expressed fewer receptors than brain glioma cells or glioma stem cells.

### Transcytosis across the BBB and effects on brain glioma and glioma stem cells

**Figure 3:**
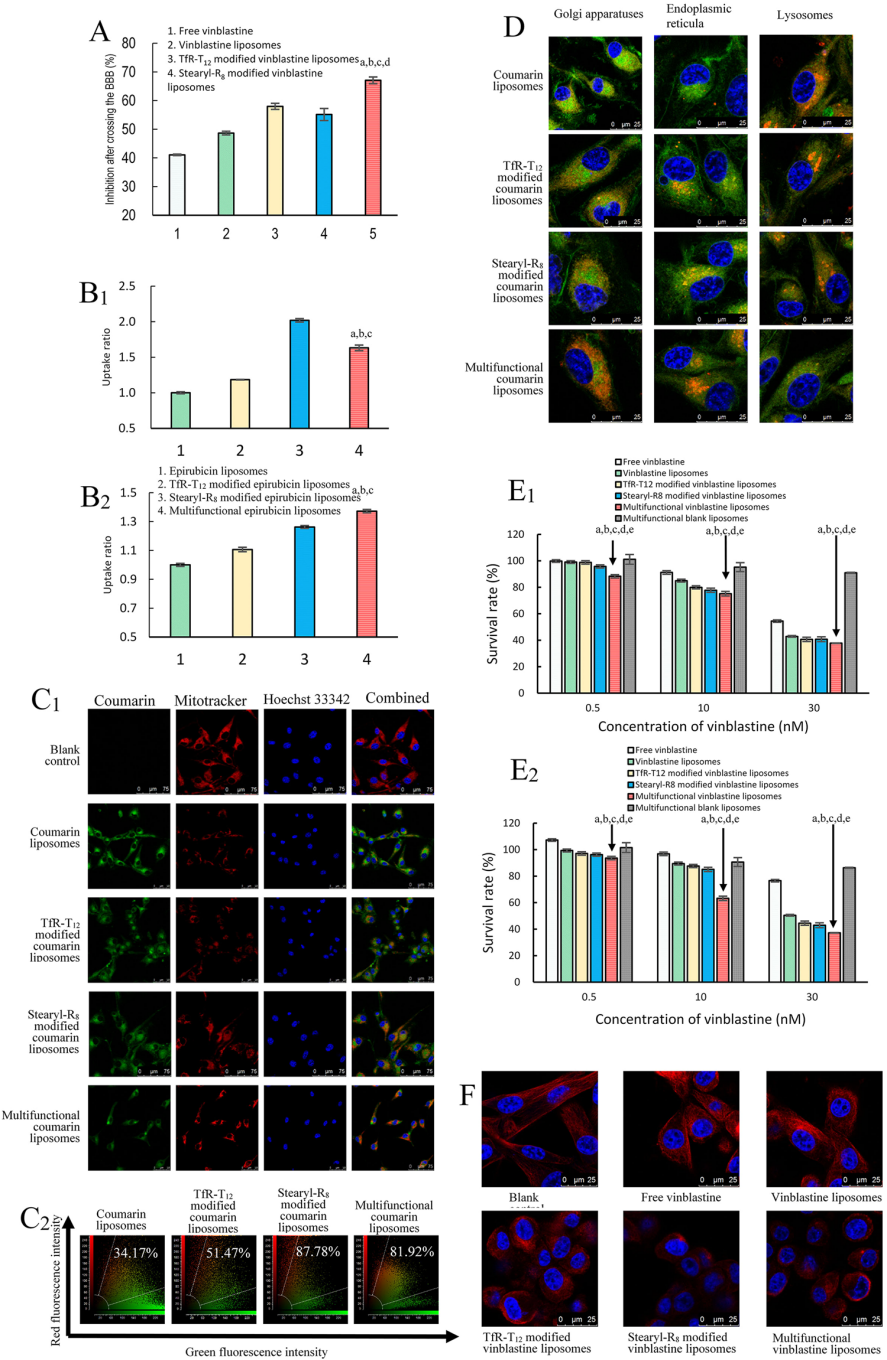
Transport across the BBB, cellular uptake and co-localization in organelles and cytotoxicity after treatment with various formulations. (**A**) Anti-glioma effect after transport across the BBB in the co-culture BBB models. *P* < 0.05; a, vs. 1; b, vs. 2; c, vs. 3; d, vs. 4. (**B**) Uptake ratios of drugs in brain glioma cells (**B**_**1**_) and glioma stem cells (**B**_**2**_). *P* < 0.05; a, vs. 1; b, vs. 2; c, vs. 3. (**C**) Co-localization in mitochondria of brain glioma cells: co-localization images (**C**_**1**_) and quantitative analysis of co-localization rates (**C**_**2**_). (**D**) Co-localization in Golgi apparatuses, endoplasmic reticula and lysosomes of brain glioma cells. Data are presented as the means ± standard deviation (n = 3). (**E**) Survival rates of brain glioma (**E**_**1**_) and glioma stem cells (**E**_**2**_) after treatments with varying drug formulations. Data are presented as the means ± standard deviation (n = 3). *P* < 0.05; a, vs. free vinblastine; b, vs. vinblastine liposomes; c, vs. TfR-T_12_ modified vinblastine liposomes; d, vs. stearyl-R_8_ modified vinblastine liposomes; e, vs. multifunctional blank liposomes. (**F**) Destruction of microtubules in brain glioma cells.

Transcytosis was performed using the *in vitro* co-culture BBB model. Trans-epithelial electrical resistance (TEER) values were used to monitor the integrity of the BBB through the experiment, and TEER values above 300 Ω·cm^2^ indicated a successful establishment of the *in vitro* BBB model. In this model, brain glioma cells containing GSCs were grown in the lower compartment of the BBB insert. Accordingly, after drug treatments were applied to the upper compartment of the BBB insert, the inhibition rates of the brain glioma cells indicated the transport capability of the multifunctional vinblastine liposomes across the BBB. The results showed the following ranking for transport across the BBB: multifunctional vinblastine liposomes > TfR-T_12_ modified vinblastine liposomes > stearyl-R_8_ modified vinblastine liposomes > vinblastine liposomes > free vinblastine (shown in inhibition rates, Fig. 3A).

The uptakes by brain glioma and by glioma stem cells were assayed by flow cytometry. After drug treatment, fluorescence intensities were used to indicate the cellular uptake either by brain glioma cells (Fig. 3B_1_) or by glioma stem cells (Fig. 3B_2_). The results showed the following ranking for the uptake ratios by brain glioma cells: stearyl-R_8_ modified epirubicin liposomes (2.02 ± 0.02) > multifunctional epirubicin liposomes (1.63 ± 0.04) > TfR-T_12_ modified epirubicin liposomes (1.18 ± 0.01) > epirubicin liposomes. In glioma stem cells, the following ranking was observed: multifunctional epirubicin liposomes (1.37 ± 0.01) > stearyl-R_8_ modified epirubicin liposomes (1.26 ± 0.01) > TfR-T_12_ modified epirubicin liposomes (1.11 ± 0.02) > epirubicin liposomes.

Co-localization in mitochondria was evaluated by confocal laser scanning fluorescence microscopy. The confocal images were intuitively used to observe the co-localization of fluorescent probe labeled multifunctional liposomes in the mitochondria (Fig. 3C_1_), and the quantitative co-localization rates were calculated by evaluating the overlapping between the red (mitotracker) and green (coumarin) fluorescence (Fig. 3C_2_). The results showed the following ranking for co-localization rates in mitochondria: stearyl-R_8_ modified coumarin liposomes (87.78%) > multifunctional coumarin liposomes (81.92%) > TfR-T_12_ modified coumarin liposomes (51.47%) > coumarin liposomes (34.17%).

The co-localization in Golgi apparatuses, endoplasmic reticula and lysosomes were evaluated by confocal laser scanning fluorescence microscopy in which the drug distributions in brain glioma were observed. The following ranking for the accumulation in the Golgi or the endoplasmic reticula was observed: multifunctional coumarin liposomes > stearyl-R_8_ modified coumarin liposomes ≥ TfR-T_12_ modified coumarin liposomes > coumarin liposomes. The ranking for the accumulation in lysosomes was coumarin liposomes > stearyl-R_8_ modified coumarin liposomes ≥ TfR-T_12_ modified coumarin liposomes > multifunctional coumarin liposomes. These results showed that the multifunctional coumarin liposomes accumulated the most in the Golgi and the endoplasmic reticula but the least in lysosomes (Fig. 3D).

The cytotoxicity of brain glioma cells or glioma stem cells after treatment with varying formulations was evaluated (Fig. 3E_1,2_). The ranking of inhibition effects on brain glioma or glioma stem cells was as follows: multifunctional vinblastine liposomes > stearyl-R_8_ modified vinblastine liposomes ≥ TfR-T_12_ modified vinblastine liposomes > vinblastine liposomes > free vinblastine > multifunctional blank liposomes. Multifunctional vinblastine liposomes had the strongest killing effects against both brain glioma and glioma stem cells.

The destruction of microtubules was evaluated by confocal laser scanning fluorescence microscopy (Fig. 3F). The microtubules were labeled with red fluorescence. The results showed the following ranking of microtubules destruction effects in brain glioma: multifunctional vinblastine liposomes ≥ stearyl-R_8_ modified vinblastine liposomes > TfR-T_12_ modified vinblastine liposomes > vinblastine liposomes > free vinblastine > multifunctional blank liposomes.

### Induction of apoptosis, autophagy and associated mechanisms

**Figure 4:**
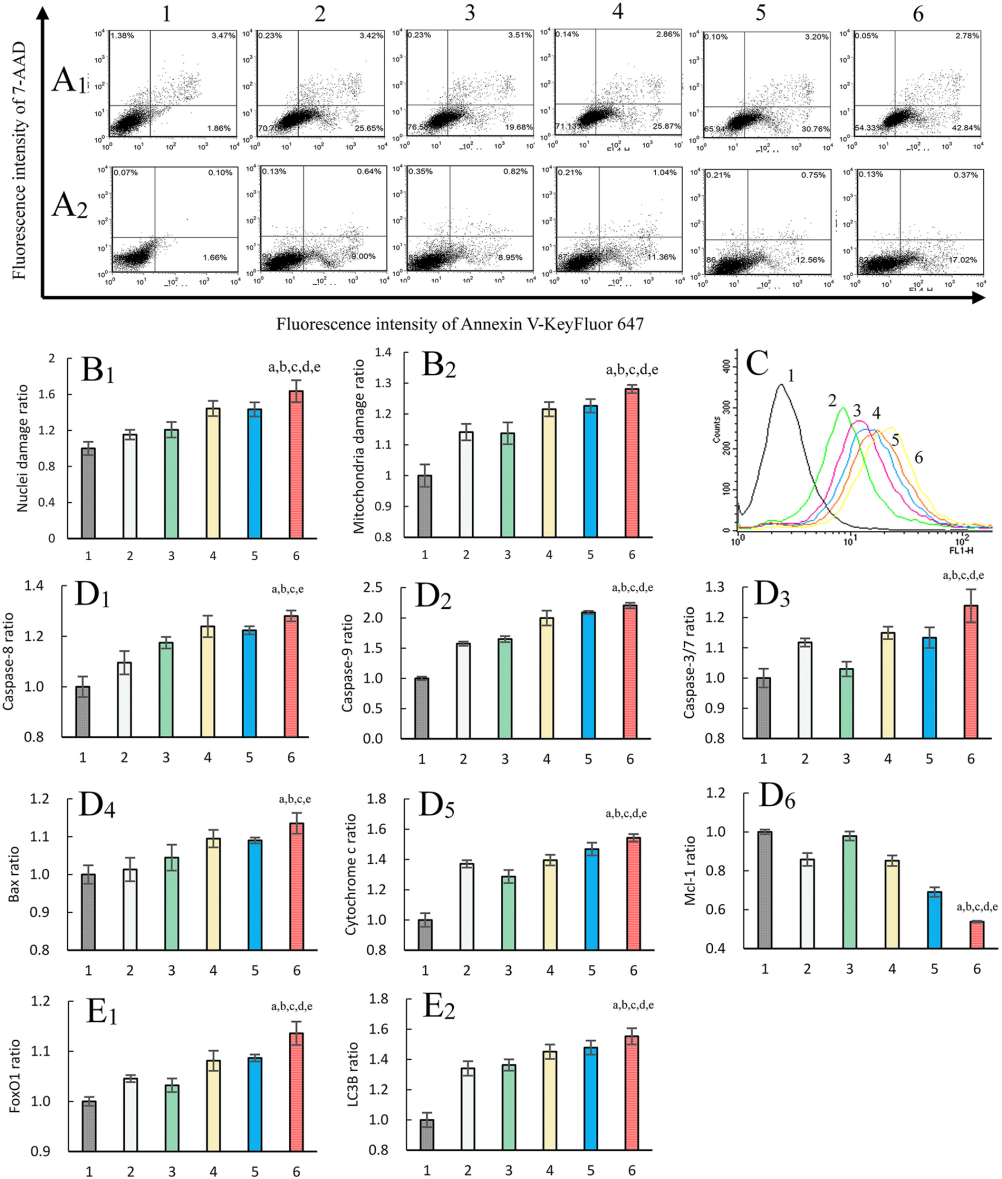
Mechanisms for induction of necrosis, apoptosis and autophagy by multifunctional vinblastine liposomes. (**A**) Apoptosis induction effects on brain glioma cells (**A**_**1**_) and glioma stem cells (**A**_**2**_). (**B**) Damage ratios of nuclei (**B**_**1**_) and mitochondria (**B**_**2**_). (**C**) Regulation of reactive oxygen species level. (**D**) Activating upstream apoptotic enzyme caspase-8 (**D**_**1**_), caspase-9 (**D**_**2**_) and downstream caspase-3/7 (**D**_**3**_), upregulating the expression of proapoptotic protein Bax (**D**_**4**_), and releasing cytochrome c (**D**_**5**_) while downregulating the expression of anti-apoptotic protein Mcl-1 (**D**_**6**_). (**E**) Upregulating the expression of autophagic proteins FoxO1 (**E**_**1**_) and LC3B (**E**_**2**_). 1, blank control; 2, free vinblastine; 3, vinblastine liposomes; 4, TfR-T_12_ modified vinblastine liposomes; 5, stearyl-R_8_ modified vinblastine liposomes; 6, multifunctional vinblastine liposomes. Data are presented as the means ± standard deviation (n = 6). *P* < 0.05; a, vs. 1; b, vs. 2; c, vs. 3; d, vs. 4; e, vs. 5.

The induction of apoptosis in brain glioma cells (Fig. 4A_1_) and glioma stem cells (Fig. 4A_2_) was evaluated by flow cytometry. The results showed that multifunctional vinblastine liposomes most strongly induced apoptosis effects in both brain glioma cells and glioma stem cells compared to the control groups.

The damage to nuclei and mitochondria of brain glioma cells were evaluated and quantified using the Operetta high content screening system. The damage ratios for nuclei (Fig. 4B_1_) and mitochondria (Fig. 4B_2_) were as follows: free vinblastine (1.15 ± 0.05; 1.14 ± 0.03), vinblastine liposomes (1.21 ± 0.08; 1.14 ± 0.04), TfR-T_12_ modified vinblastine liposomes (1.44 ± 0.08; 1.22 ± 0.02), stearyl-R_8_ modified vinblastine liposomes (1.43 ± 0.08; 1.23 ± 0.02), multifunctional vinblastine liposomes (1.64 ± 0.12; 1.28 ± 0.01). Multifunctional vinblastine liposomes produced the most significant damage to both nuclei and mitochondria.

The induced reactive oxygen species (ROS) levels in brain glioma were evaluated by flow cytometry (Fig. 4C). After drug treatments, the generated ROS levels were indicated by the fluorescence intensity of dichlorofluorescein (DCF), which is a product of reactive oxygen species (ROS)-induced oxidation of 2,7-dichlorofluorescein diacetate (DCFH-DA). The results showed the following ranking for ROS induction: multifunctional vinblastine liposomes > stearyl-R_8_ modified vinblastine liposomes > TfR-T_12_ modified vinblastine liposomes > vinblastine liposomes > free vinblastine > blank control.

The signaling pathways related to apoptosis and autophagy were evaluated by assaying the changes in the related enzymes or proteins using the Operetta high content screening system. After treatment with free vinblastine, vinblastine liposomes, TfR-T_12_ modified vinblastine liposomes, stearyl-R_8_ modified vinblastine liposomes or multifunctional vinblastine liposomes, the respective expression ratios of caspase-8 (Fig. 4D_1_) were 1.10 ± 0.05, 1.17 ± 0.02, 1.24 ± 0.04, 1.22 ± 0.02, and 1.28 ± 0.02; the expression ratios of caspase-9 (Fig. 4D_2_) were 1.57 ± 0.03, 1.65 ± 0.05, 2.00 ± 0.12, 2.09 ± 0.03, 2.20 ± 0.04; the expression ratios of caspase-3/7 (Fig. 4D_3_) were 1.12 ± 0.01, 1.03 ± 0.02, 1.15 ± 0.02, 1.13 ± 0.03, 1.24 ± 0.05; the expression ratios of Bax (Fig. 4D_4_) were 1.01 ± 0.03, 1.04 ± 0.03, 1.09 ± 0.02, 1.09 ± 0.01, 1.14 ± 0.03; the expression ratios of cytochrome c (Fig. 4D_5_) were 1.37 ± 0.02, 1.29 ± 0.04, 1.40 ± 0.04, 1.47 ± 0.04, 1.54 ± 0.02; the expression ratios of Mcl-1 (Fig. 4D_6_) were 0.86 ± 0.03, 0.98 ± 0.02, 0.85 ± 0.03, 0.69 ± 0.02, 0.54 ± 0.01; the expression ratios of Forkhead box protein O1 (FoxO1) (Fig. 4E_1_) were 1.05 ± 0.01, 1.03 ± 0.01, 1.08 ± 0.02, 1.09 ± 0.01, 1.14 ± 0.02; and the expression ratios of light chain 3B (LC3B) (Fig. 4E_2_) were 1.34 ± 0.05, 1.36 ± 0.04, 1.45 ± 0.05, 1.48 ± 0.05, 1.55 ± 0.05.

### Treatment of brain glioma and glioma stem cells in mice

**Figure 5:**
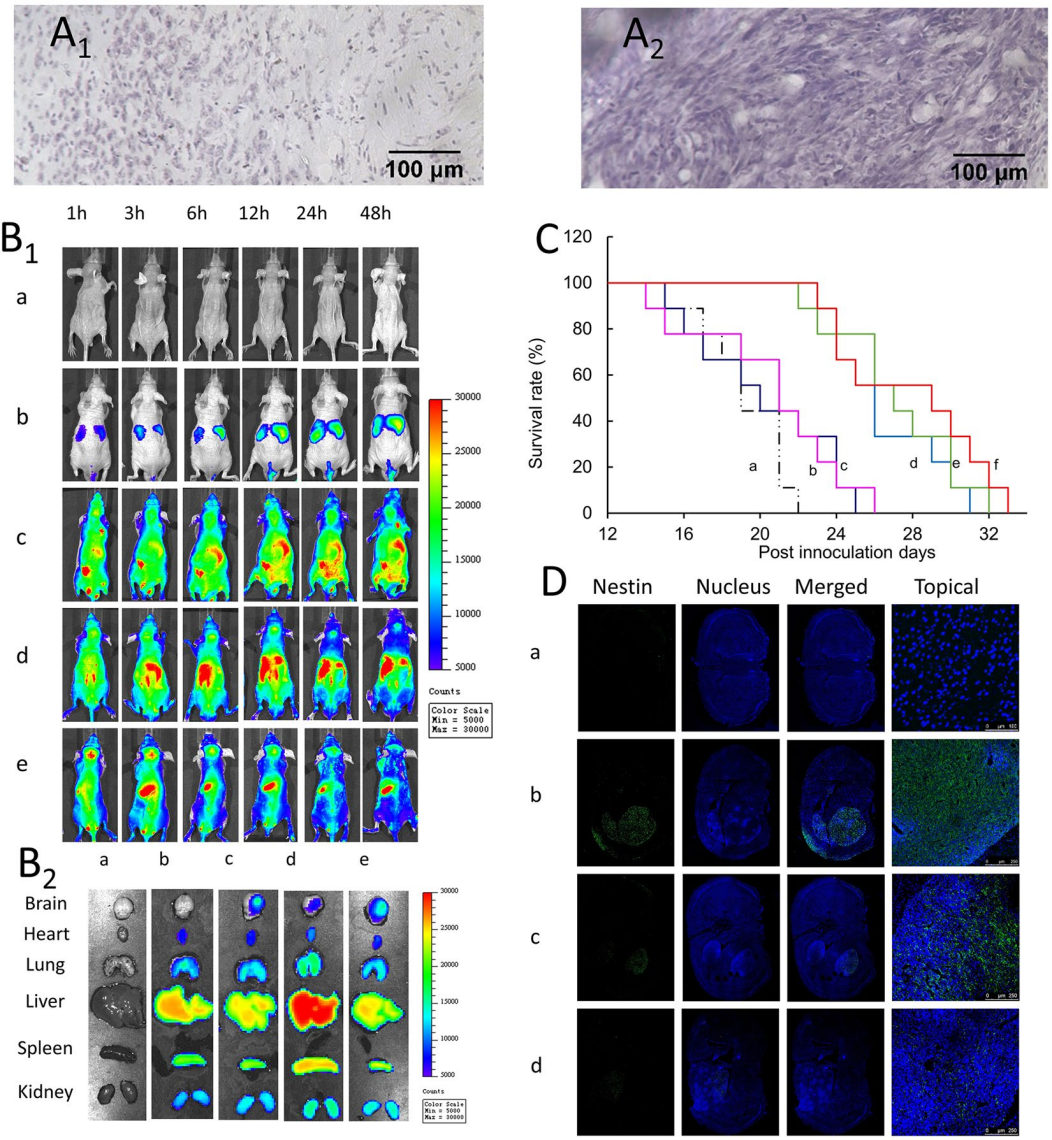
Anticancer efficacy in brain glioma-bearing mice after intravenous administration of multifunctional vinblastine liposomes. (**A**) Nissl staining of the normal brain (**A**_**1**_) and pathological brain (**A**_**2**_). (**B**) *In vivo* real-time imaging (**B**_**1**_) and *ex vivo* imaging at 48 h (**B**_**2**_) for observing the distributions: a, physiological saline; b, free DiR; c, TfR-T_12_ modified DiR liposomes; d, stearyl-R_8_ modified DiR liposomes; e, multifunctional DiR liposomes. (**C**) Kaplan-Meier survival curves of brain glioma-bearing mice after treatment with varying formulations: a, physiological saline; b, free vinblastine; c, vinblastine liposomes; d, TfR-T_12_ modified vinblastine liposomes; e, stearyl-R_8_ modified vinblastine liposomes; f, multifunctional vinblastine liposomes. (**D**) Anti-stem cells efficacy *in vivo*: a, normal brain; b, brain glioma after treatment with physiological saline; c, brain glioma after treatment with vinblastine liposomes; d, brain glioma after treatment with multifunctional vinblastine liposomes.

The brain glioma-bearing mouse model was validated by Nissl staining of normal brains (Fig. 5A_1_) and pathological brains (Fig. 5A_2_). The result showed that the cells in the normal brain section were sparsely distributed, whereas those in the pathological brain section were densely distributed, indicating that the establishment of the brain glioma-bearing mice model was successful.

The real-time distribution in brain glioma-bearing nude mice after intravenous administration of fluorescent probe labeled multifunctional liposomes was observed using the *in vivo* imaging system (Fig. 5B_1_). The results showed that compared to the controls, multifunctional DiR liposomes exhibited the strongest fluorescence intensity in the brain. In contrast, the free DiR group showed no fluorescence signal in brain tissue.

The mice were sacrificed at 48 h after drug administration and the brain, heart, liver, spleen, lung, and kidney tissues were isolated for *ex vivo* imaging (Fig. 5B_2_). The results demonstrated that the free DiR liposomes exhibited no distribution in the brain but accumulated in the other tissues. In contrast, the multifunctional DiR liposomes demonstrated the most significant accumulation in the brain tissues compared to the controls. Notably, the liposomal formulations were also distributed in the other tissues.

The overall efficacy of the treatment of brains in mice was evaluated using the brain glioma-bearing nude mice. The results showed the following ranking for survival days (Fig. 5C, and Supplementary Table S2) after intravenous administration: multifunctional vinblastine liposomes (29 days, increased by 52.63% comparing to physiological saline) > stearyl-R_8_ modified vinblastine liposomes (27 days, 42.11%) > TfR-T_12_ modified vinblastine liposomes (26 days, 36.84%) > vinblastine liposomes (21 days, 10.53%) > free vinblastine (20 days, 5.26%) > physiological saline (19 days).

The elimination effect of GSCs in the brain glioma-bearing mice was evaluated by observing the brain sections in which nestin was used as biomarker of glioma stem cells (Fig. 5D). The glioma stem cell quantities detected in the sections was ranked as follows: physiological saline > vinblastine liposomes > multifunctional vinblastine liposomes. These results indicated that the multifunctional vinblastine liposomes had the strongest efficacy in eliminating glioma stem cells.

## Discussion

Brain glioma is a severe malignant disease, and its treatment remains an unsolved problem. Brain gliomas may grow evenly in the contralateral hemisphere, can hide within cranial bones, and are protected by the BBB. A complete surgical removal of glioma is virtually impossible. Furthermore, surgical operation and/or radiotherapy cannot fully eliminate brain glioma and glioma stem cells, and relapse is inevitable. Thus, chemotherapy continues to play an important role in the treatment of brain glioma. However, because the BBB and glioma stem cells are the major obstacles to chemotherapy, the exploration new chemotherapeutic strategies are urgently needed. In this study, a type of multifunctional drug lipid vesicles was developed by modifying the liposomes with two functional materials, TfR-T_12_-PEG_2000_-DSPE and stearyl-R_8_. The constructed multifunctional vinblastine liposomes could be transported across the BBB by RMT and AMT and specifically entered brain glioma and glioma stem cells via receptor mediated endocytosis (RME) and adsorptive mediated endocytosis (AME), thereby eliminating brain glioma and glioma stem cells by triggering their necrosis, apoptosis and autophagy.

Vinblastine is a chemotherapeutic drug used to treat a variety of cancers, and it takes effect by inhibiting the assembly of microtubules and interrupting the formation of the mitotic spindle and kinetochore, thereby killing cancer cells^20, 21^. Vinblastine is included as a model drug because it is very effective in killing brain glioma *in vitro* but is ineffective *in vivo* because of the BBB. However, like most cell cycle-specific anticancer agents, vinblastine also has minimal effects on glioma stem cells because of the common features of cancer stem cells^22^.

To overcome these problems, the multifunctional vinblastine liposomes were fabricated to transfer vinblastine across the BBB and increase its internalization by both brain glioma and glioma stem cells. Besides, the multifunctional lipid vesicles strongly potentiated the effects of vinblastine in triggering apoptosis and autophagy of brain glioma and/or glioma stem cells as well, in addition to their necrosis as a usual way.

In multifunctional vinblastine liposomes, the newly synthesized TfR-T_12_-PEG_2000_-DSPE acts as a BBB-targeting molecule that specifically binds to transferrin receptors, facilitates the ferrying of the liposomes across the BBB by RMT, and allows the liposomes to penetrate brain glioma cells and glioma stem cells via RME. Furthermore, the PEG_2000_-DSPE moiety confers the long circulatory function of the liposomes in the blood by allowing the vesicles to escape the rapid uptake of the reticuloendothelial system (RES)^23^. The cationic peptide conjugate stearyl-R_8_ (stearyl-RRRRRRRR) plays a role in helping achieve AMT and AME because the lipophilic stearyl group can be inserted into the bilayer of liposomes (Fig. 1B), whereas R_8_ with its eight arginines with multi-cationic centers is left outside of the liposome vesicles, enabling the liposomes to target mitochondria easily in response to their negatively charged potential^24^. Moreover, a lower drug release rate in the initial 24 h (Fig. 1D) indicated less drug leakage in the blood circulation after intravenous administration. In addition to these findings, the particle size of the liposomes (Supplementary Table S1) would be suitable and beneficial for the enhanced permeability and retention (EPR) effect in brain glioma^25^.

Although the biomarkers for identifying cancer stem cells remain controversial, nestin (Fig. 2A_1,2_) is considered as a characteristic marker for identifying glioma stem cells^26^. ABCG2 transporter is also considered a marker because of its higher expression on glioma stem cells compared with brain glioma cells (Fig. 2B_1,2_)^27^. Chemokine receptor type 4 (CXCR4) has also been identified as a marker (Fig. 2C_1,2_) because it is highly expressed in the CD133^+^ brain glioma side population cells^28^. Therefore, nestin, ABCG2 and CXCR4 are included as the phenotype markers for identifying glioma stem cells.

The expression ratios of transferrin receptors on BMVECs, brain glioma cells and glioma stem cells were detected at the protein levels. The results demonstrated that the TfRs are highly expressed on brain glioma cells and glioma stem cells, whereas they are moderately expressed on BMVECs (Fig. 2D_1–3_)^29^. Accordingly, TfRs expressed on these cells could be useful ferrying sites for transporting the multifunctional vinblastine liposomes.

The transcytosis of multifunctional vinblastine liposomes was evaluated using the constructed co-culture BBB model. In the co-culture BBB model, BMVECs proliferate and form tight conjunctions, resulting in an elevated TEER. The inhibition rates of brain glioma cells in the BBB model reflect the transcytosis across the BBB. In view of the results (Fig. 3A), multifunctional vinblastine liposomes exhibited an obvious transport capability across the BBB and then a significant killing effect against brain glioma cells. Most likely, these mechanisms are associated with RMT-mediated transcytosis because of the specific interaction of TfR-T_12_ with TfRs on BMVECs in the BBB model. Moreover, the transcytosis is also caused by AMT because of the interaction of cationic R_8_ with the negatively charged BMVECs on the model^30^.

The internalization observed in the cellular uptake assay demonstrated that both TfR-T_12_-PEG_2000_-DSPE and stearyl-R_8_ contribute to promoting the cellular uptake of the drug-loaded liposomes (Fig. 3B_1,2_). The mechanisms are also related to the RME because of the highly expressed TfRs on both brain glioma cells and glioma stem cells. Moreover, the negatively charged surfaces of brain glioma cells and glioma stem cells attract the cationic stearyl-R_8_ modified drug liposomes, thereby mediating the increased cellular uptake by AME.

To a large extent, the distribution of the drug inside the cells determines how the drug takes effect. The distributions in organelles as observed by confocal microscopy indicated that the fluorescent probe-labeled multifunctional liposomes primarily accumulate in mitochondria (Fig. 3C_1,2_), partially accumulate in endoplasmic reticula and Golgi apparatuses, and scarcely accumulate in lysosomes (Fig. 3D). These phenomena demonstrate that the liposomes captured by lysosomes of brain glioma cells either can be rapidly released or can quickly escape because of the proton sponge effect, in which the cationic stearyl-R_8_ on the liposomes bind a large amount of protons and promote osmotic swelling of the lysosomes^31^. Afterwards, most of the liposomes are adsorbed onto the negatively charged mitochondria because of the modification by cationic stearyl-R_8_.

In the cytotoxicity assay, the *in vitro* effects of multifunctional vinblastine liposomes were evidenced in both brain glioma cells and glioma stem cells (Fig. 3E_1,2_). Although free vinblastine or regular vinblastine liposomes are effective in treating brain glioma, they are less effective in killing glioma stem cells. Improved efficacy primarily arises from increased endocytosis through RME mediated by TfR-T_12_-PEG_2000_-DSPE and AME mediated by the cationic material stearyl-R_8_.

The fluorescently labeled microtubules showed that the multifunctional vinblastine liposomes can cause obvious destruction to microtubules, leading to evident necrosis of brain glioma cells. In contrast, free vinblastine or regular vinblastine liposomes result in moderate destruction of the microtubules (Fig. 3F). Because vinblastine kills glioma cells primarily by inhibiting the assembly of microtubules, the increased endocytosis of vinblastine by both RME and AME results in the potentiated destruction of microtubules.

In addition to necrosis, multifunctional vinblastine liposomes kill brain glioma cells and glioma stem cells by apoptosis (Fig. 4A_1,2_), leading to obvious damage to both nuclei (Fig. 4B_1_) and mitochondria (Fig. 4B_2_). The mechanism of apoptosis is associated with the activation of apoptotic enzyme caspase-8 (Fig. 4D_1_) and caspase-9 (Fig. 4D_2_), the up-regulation of pro-apoptotic protein Bax (Fig. 4D_4_), the down-regulation of the anti-apoptotic protein Mcl-1 (Fig. 4D_6_), the induced release of cytochrome c from mitochondria (Fig. 4D_5_), the induced generation of ROS (Fig. 4C), and the subsequent activation of downstream caspase-3 (Fig. 4D_3_) during a cascade of apoptotic reactions^32^.

Furthermore, the multifunctional vinblastine liposomes result in programmed cell death via autophagy. The mechanism is related to the increased binding of Beclin-1 with class III phosphatidylinositol-3 kinase VPS34, the activation of LC3 ubiquitin-like (UBL) system^33^, the formation of FoxO1 (Fig. 4E_1_), and the transformation of LC3 to LC3B (Fig. 4E_2_).

The efficacy of multifunctional vinblastine liposomes was further demonstrated in brain glioma-bearing nude mice. The real-time imaging and *ex vivo* imaging results indicated that the fluorescent probe labeled multifunctional liposomes accumulated more in the brain glioma sites (Fig. 5B_1,2_). The overall efficacy of brain glioma treatment in mice further revealed an increased survival time after the intravenous administration of multifunctional vinblastine liposomes (Fig. 5C). Finally, the results from the immunofluorescence assay also evidenced that the liposomes had a killing effect on glioma stem cells in the brain glioma-bearing mice (Fig. 5D).

## Methods

### Synthesis of TfR-T_12_-PEG_2000_-DSPE

To synthesize TfR-T_12_-PEG_2000_-DSPE, TfR-T_12_ (THRPPMWSPVWP, 8 μmol) and NHS-PEG_2000_-DSPE (8 μmol) were dissolved in DMF (2 mL), and N-methylmorpholine (200 μL) was added into the mixture. The mixture was stirred using a magnetic stirrer at room temperature for 48 h under nitrogen gas protection. Then, the crude product was transferred into a regenerated cellulose dialysis tubing (MWCO, 3000 Da) and dialyzed against deionized water for 24 h to remove unreacted raw materials and dimethyl formamide (DMF). The mixture was freeze dried, and the product was obtained. The product was confirmed by MALDI-TOF-MS (Shimadzu, Japan).

### Fabrication and characterization of the liposomes

Four types of liposomes were prepared, including vinblastine liposomes, TfR-T_12_ modified vinblastine liposomes, stearyl-R_8_ modified vinblastine liposomes, and multifunctional vinblastine liposomes. To prepare multifunctional vinblastine liposomes, egg phosphatidylcholine (EPC), cholesterol, TfR-T_12_-PEG_2000_-DSPE and stearyl-R_8_ were dissolved in dichloromethane at a molar ratio of 60:30:3:5 in a pear-shaped bottle. The solvent was then removed using a rotary vacuum evaporator, and the lipid film was hydrated with ammonium sulfate solution by sonication (250 mM, 5 mL) in a water bath for 5 min. Afterwards, the suspensions were treated with an ultrasonic cell disruptor for 10 min (200 w), transferred into a regenerated cellulose dialysis tubing (MWCO, 8000–14,000 Da), and dialyzed in HEPES buffer solution (HBS, 25 mM HEPES, 150 mM NaCl) for 24 h. The dialyzed suspensions were incubated with vinblastine solution (lipids: drug = 20:1, w/w) in a water bath at 60 °C for 20 min with continuous shaking. Thus, the multifunctional vinblastine liposomes were obtained. Other types of liposomes were prepared accordingly.

The liposomes were observed using an atomic force microscope (SPI3800N series SPA-400, NSK Ltd., Tokyo, Japan), and measured using a Nano Series Zenith 4003 Zetasizer (Malvern Instruments Ltd., Malvern, UK). The content of vinblastine was measured by a high-performance liquid chromatography system (Shimadzu, Japan). The mobile phase for measuring vinblastine consisted of methanol and 0.02 M NaH_2_PO_4_ (70:30, v/v), and the detection wavelength was set at 267 nm.

*In vitro* release rates of vinblastine in the liposomes were determined by dialysis against phosphate buffered saline (PBS, 137 mM NaCl, 2.7 mM KCl, 8 mM Na_2_HPO_4_ and 2 mM KH_2_PO_4_, pH 7.4) containing 10% FBS.

### Animal experiments

All of the animal experiments adhered to the principles of care and use of laboratory animals and were approved by the Institutional Animal Care and Use Committee of Peking University.

(Please refer to *Supplementary data* for more details of methods).

## Conclusions

**Figure 6:**
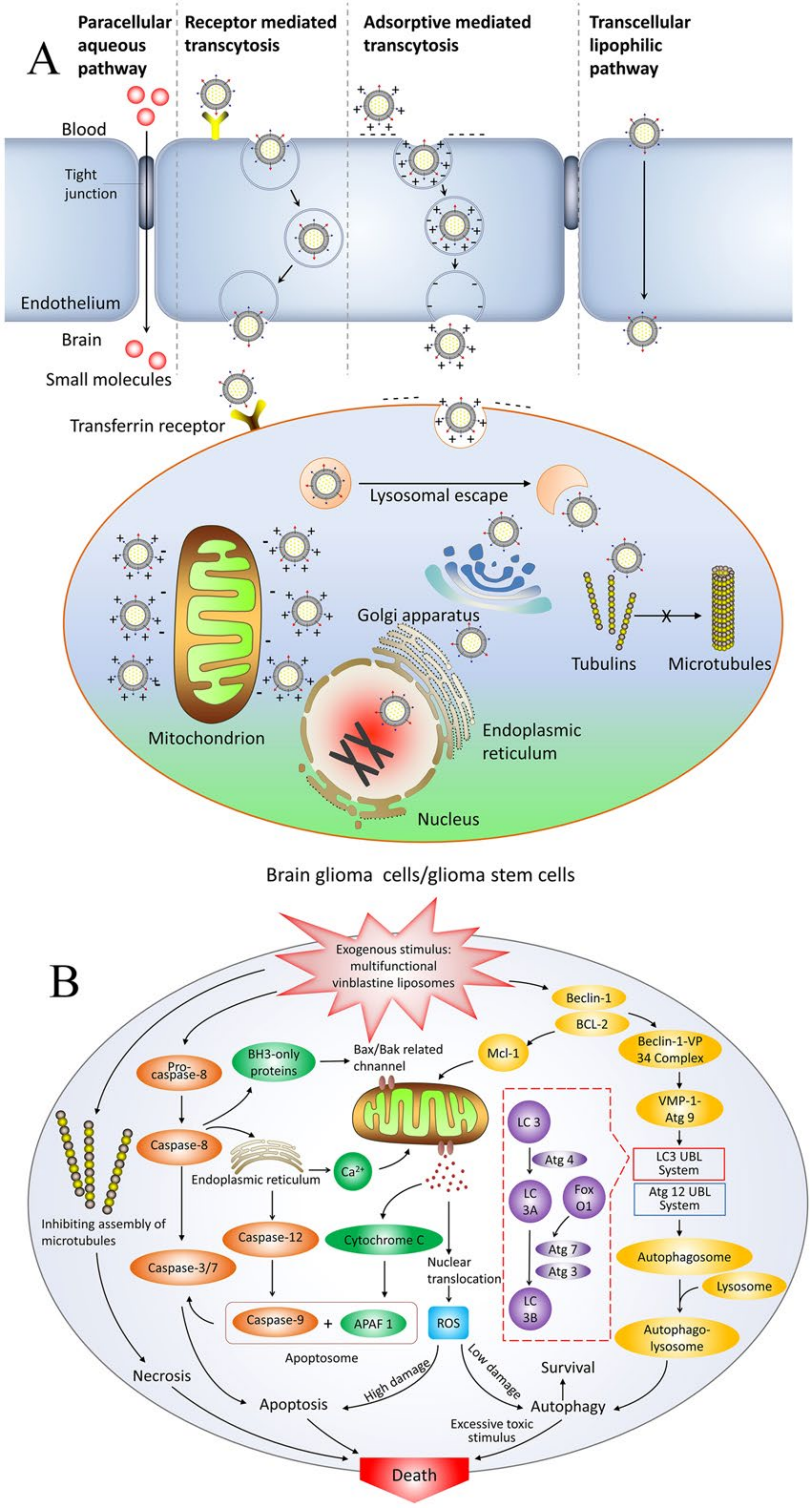
Schematic representations of transcytosis of multifunctional vinblastine liposomes across the BBB and elimination of brain glioma and glioma stem cells. (**A**) Multifunctional vinblastine liposomes are transported across the blood-brain barrier, enter brain glioma or glioma stem cells, and are distributed throughout the cells. (**B**) The signaling pathways for necrosis, apoptosis and autophagy in brain glioma or glioma stem cells are triggered by multifunctional vinblastine liposomes.

In conclusion, a novel type of multifunctional vinblastine liposomes was fabricated by modifying a transferrin peptide-lipid conjugate (TfR-T_12_-PEG_2000_-DSPE) and a cationic stearylated oligo-peptide (stearyl-R_8_). The liposomes are approximately 110 nm and are long-circulating in the blood. They are able to transport across the BBB through RMT and AMT, followed by a potentiated endocytosis by both brain glioma cells and glioma stem cells through RME and AME (Fig. 6A). The liposomes exhibit significant efficacy against brain glioma and glioma stem cells both *in vitro* and *in vivo*. The action mechanism on brain glioma or glioma stem cells is related to three aspects: improved cellular uptake leads to increased necrosis by inhibiting the assembly of microtubules; apoptosis is induced by the activation of apoptotic enzyme caspase-8, 9 and 3/7, the up-regulation of pro-apoptotic protein Bax, the down-regulation of anti-apoptotic protein Mcl-1, the release of cytochrome c from mitochondria, and the induced generation of ROS; and autophagy is induced by the activation of the LC3 ubiquitin-like (UBL) system via the formation of FoxO1 and the transformation of LC3 to LC3B (Fig. 6B). Therefore, multifunctional vinblastine liposomes could offer a new strategy for the treatment of brain glioma and glioma stem cells.

## Electronic supplementary material

Supplementary information
