# Supplementary material for: Lipid vesicles containing transferrin receptor binding peptide TfR-T12 and octa-arginine conjugate stearyl-R8 efficiently treat brain glioma along with glioma stem cells
Source: Sci Rep. 2017 Jun 14;7:3487. doi: 10.1038/s41598-017-03805-7 (PMC5471209; doi:10.1038/s41598-017-03805-7)
Supplement: Supplementary file 1 — Supplementary information [file 41598_2017_3805_MOESM1_ESM.pdf]

Supplementary methods, tables and figures

Lipid vesicles containing transferrin receptor binding peptide TfR-T_{12} and octa-arginine conjugate stearyl-R_8 efficiently treat brain glioma along with glioma stem cells

Li-Min Mu[1], Ying-Zi Bu[1], Lei Liu[1], Hong-Jun Xie[1], Rui-Jun Ju[1,2], Jia-Shuan Wu[1], Fan Zeng[1], Yao Zhao[1], Jing-Ying Zhang[1], Wan-Liang Lu[1,*]

[1]Beijing Key Laboratory of Molecular Pharmaceutics and New Drug System, State Key Laboratory of Natural and Biomimetic Drugs, and School of Pharmaceutical Sciences, Peking University, Beijing, China

[2]Department of Pharmaceutical Engineering, Beijing Institute of Petrochemical Technology, Beijing, China

***Corresponding author:**

Wan-Liang Lu, Ph.D.

Professor and Vice Dean

School of Pharmaceutical Sciences

Peking University

Beijing 100191, China

E-mail: luwl@bjmu.edu.cn

Tel.: +8610 82802683

Fax: +8610 82802683

Materials and methods

Materials and cells

Vinblastine was purchased from Nanjing Duolun Chemicals (Nanjing, China), PEG_{2000}-DSPE and NHS-PEG_{2000}-DSPE were purchased from NOF Corporation (Kanagawa, Japan), TfR-T_{12} peptide was synthesized by Hefei Bankpeptide Company Ltd. (Hefei, China), and stearyl-R_8 peptide was from Shanghai GL Biochem Ltd. (Shanghai, China).

Murine brain microvascular endothelial cells (BMVECs) were obtained from the Institute of Clinical Medical Sciences, China-Japan Friendship Hospital (Beijing, China) and were cultured in Dulbecco's modified eagle medium (DMEM) supplemented with 20% fetal bovine serum (FBS), 100 U/mL penicillin, 100 μg/mL streptomycin, 40 U/mL heparin and 100 ug/mL endothelial cell growth factor.

Brain glioma cells were purchased from Institute of Basic Medical Science, Chinese Academy of Medical Science (Beijing, China) and were cultured in minimum essential medium eagle (MEM) supplemented with 10% FBS and 1% non-essential amino acids (Macgene, Beijing, China).

Glioma stem cells were induced and cultured in DMEM-F12 medium supplemented with 2% B27 supplements (Gibco), epidermal growth factor (EGF, 20 ng/mL), basic fibroblast growth factor (bFGF, 20 ng/mL), leukemia inhibitory factor (LIF, 10 ng/mL) and insulin (181.6 ng/mL). All cells were cultured under an atmosphere of 5% CO_2 at 37 °C. Other materials used were commercially available.

Identification of glioma stem cells

The phenotypes of the induced glioma stem cells were identified by flow cytometry

(FCM), including nestin, ABCG2 and CXCR4. Briefly, brain glioma cells and glioma stem cells were collected and perforated using 0.2% Triton X-100 solution. Then, the cells were incubated for 30 min at room temperature with anti-human nestin fluorescein isothiocyanate (FITC)-conjugated antibody (R&D, 10 μL per test). The cells were washed with PBS twice and re-suspended in 300 μL PBS, and the fluorescence intensity was measured by FCM.

To identify ABCG2 and CXCR4, anti-human ABCG2 FITC-conjugated antibody (BioLegend, Beijing, China; 5 μL per test) and anti-human CXCR4 phycoerythrin (PE)-conjugated antibody (BioLegend, Beijing, China; 5 μL per test) were separately added using the procedures described above except for the perforation.

Both brain glioma cells and glioma stem cells were incubated with FITC-conjugated mouse IgG or PE-conjugated mouse IgG as isotype controls, respectively. The excitation wavelengths were set at 488 nm for both FITC and PE, and the emission wavelengths were set at 530 nm for FITC and 564 nm for PE.

Identification of transferrin receptors

Brain microvascular endothelial cells were collected and incubated at room temperature for 20 min with anti-mouse transferrin receptors FITC-conjugated antibody (eBioscience, Beijing, China; 5 μL per test). Then, the cells were washed twice with PBS and re-suspended in 300 μL PBS. The fluorescence intensity was measured by FCM.

Brain glioma cells and glioma stem cells were treated as above, and anti-human transferrin receptors FITC-conjugated antibody (Sino Biological Inc., Beijing, China; 5 μL per test) was used.

Transport across the BBB

Brain microvascular endothelial cells were seeded in polyester membrane inserts (Corning 3460, NY, USA) incubated with 2% gelatin (Topbio, Beijing, China) in Hank's balanced salt solution (Magene, Beijing, China) at a density of 1×10^4 cells per insert. The cells were cultured for 6 days, and the culture medium was changed every two days. Then, the trans-epithelial electric resistance (TEER) was measured using Epithelial Vol-Ohm Meter (Millicell ERS-2, Millipore, Billerica, USA) to monitor the integrity of the BBB model.

Afterwards, brain glioma cells were seeded in the lower compartment at a density of 2×10^3 cells/well, and the upper compartment contained brain microvascular endothelial cells. This system was cultured for 24 h, thereby establishing the co-culture BBB model.

Drugs consisting of free vinblastine, vinblastine liposomes, TfR-T_{12} modified vinblastine liposomes, stearyl-R_8 modified vinblastine liposomes or multifunctional vinblastine liposomes (each with a concentration of 30 nM vinblastine) were added into the upper compartment. After incubation for 48 h, the inhibition rate of brain glioma cells was determined using the sulforhodamine B (SRB) staining assay.

Cellular uptake

Epirubicin was used to replace vinblastine in the four types of liposomes as the fluorescent probe to observe the cellular uptake of the liposomes. Brain glioma cells and glioma stem cells were seeded individually in 12-well plates at a density of 2×10^5 cells/well and were cultured for 24 h. Then, the culture media were replaced with fresh media containing epirubicin liposomes, TfR-T_{12} modified epirubicin liposomes, stearyl-R_8 modified epirubicin liposomes or multifunctional epirubicin liposomes, each with a concentration of 10 μM

epirubicin. After incubation for 2 h, the cells were collected and re-suspended in PBS. The fluorescence intensity was measured by FCM. The excitation wavelength was set at 488 nm and the emission wavelength was set at 560 nm.

Mitochondria targeting

Coumarin was used to replace vinblastine in the four types of liposomes as the fluorescent probe for observing the mitochondria targeting ability of the liposomes. Brain glioma cells were seeded in glass bottom dishes (Nest, Beijing, China; φ = 15 mm,) at a density of 2×10^5 cells in each dish and then cultured for 24 h. Then, the liposomes, including coumarin liposomes, TfR-T_{12} modified coumarin liposomes, stearyl-R_8 modified coumarin liposomes and multifunctional coumarin liposomes, were separately added into the bottoms of the dishes at a concentration of 100 nM coumarin; blank medium was added as blank control. After incubation for 4 h, the cells were washed with PBS and stained with MitoTracker Deep Red FM (Invitrogen, Beijing, China) and Hoechst 33342 (Invitrogen, Beijing, China) at 37 °C for 0.5 h. The mitochondrial targeting ability was evaluated using a confocal laser scanning fluorescence microscope (Leica, Heidelberg, Germany).

Distribution in brain glioma

Coumarin was used to replace vinblastine in the four types of liposomes as the fluorescent probe. Brain glioma cells were seeded in glass bottom dishes (Nest China; φ = 15 mm) at a density of 2×10^5 cells in each dish; the cells were then cultured for 24 h. Next, the liposomes, including coumarin liposomes, TfR-T_{12} modified coumarin liposomes, stearyl-R_8 modified coumarin liposomes and multifunctional coumarin liposomes, were separately added into the dishes at a concentration of 100 nM coumarin; blank medium was added as blank

control. After incubation for 4 h, the cells were washed with PBS and stained with Golgi-Tracker Red, ER-Tracker Red, and Lyso-Tracker Red (Beyotime, Beijing, China), respectively, and Hoechst 33342 (Invitrogen, Beijing, China) at 37 °C for 0.5 h. The drug distribution was evaluated using a confocal laser scanning fluorescence microscope (Leica, Heidelberg, Germany).

Cytotoxicity

Brain glioma cells or glioma stem cells were seeded in 96-well plates at a density of 4×10^3 cells/well and cultured for 24 h. Next, the culture media were replaced with fresh media containing free vinblastine, vinblastine liposomes, TfR-T_{12} modified vinblastine liposomes, stearyl-R_8 modified vinblastine liposomes or multifunctional vinblastine liposomes, with varying concentrations ranging from 0 to 50 nM vinblastine. After incubation for 48 h, the cells were fixed with 10% trichloroacetic and stained with sulforhodamine B (SRB). The absorbance of each well was measured using a microplate reader (Infinite F50, Tecan Group Ltd., Beijing, China) at a wavelength of 540 nm. Survival rate = (A_{540nm} of treated cells / A_{540nm} of blank control cells) × 100%. A_{540nm} refers to absorbance at 540 nm.

Microtubule damage

Brain glioma cells were seeded in glass bottom dishes at a density of 2×10^5 cells each and cultured for 24 h. Next, drugs, including free vinblastine, vinblastine liposomes, TfR-T_{12} modified vinblastine liposomes, stearyl-R_8 modified vinblastine liposomes and multifunctional vinblastine liposomes, were separately added at a concentrations of 30 nM vinblastine. Blank medium was added as blank control. After incubation for 3 h, the cells were washed with PBS and stained with Tubulin-Tracker Red (Beyotime, Beijing, China) and Hoechst 33342 for 0.5 h

at 37 °C. The microtubule destruction was evaluated using a confocal laser scanning fluorescence microscope.

Induction of apoptosis

Brain glioma cells or glioma stem cells were seeded in 6-well plates at a density of 5×10^5 cells/well and cultured for 24 h. Then, the culture media were separately replaced with fresh media containing free vinblastine, vinblastine liposomes, TfR-T_{12} modified vinblastine liposomes, stearyl-R_8 modified vinblastine liposomes and multifunctional vinblastine liposomes at a concentration of 30 nM vinblastine. After incubation for 12 h, the cells were collected, and stained with 7-AAD and Annexin V-KeyFluor 647 (KeyGEN Biotech, Shanghai, China) according to the product instructions. The fluorescence intensity was measured by FCM.

Nuclei and mitochondria damages

Brain glioma cells were seeded in 12-well plates at a density of 1×10^5 cells/well and were cultured for 24 h. Next, the culture media were replaced with fresh media containing free vinblastine, vinblastine liposomes, TfR-T_{12} modified vinblastine liposomes, stearyl-R_8 modified vinblastine liposomes or multifunctional vinblastine liposomes, with a concentration of 30 nM vinblastine. After incubation for 12 h, the medium was replaced with fresh medium containing MitoTracker Deep Red FM (200 nM) and Hoechst 33342 (5 μg/mL). After staining at 37 °C for 0.5 h, the cells were fixed using 4% paraformaldehyde for 10 min and washed with PBS. The images were obtained using the Operetta high content screening system (Perkin Elmer, Waltham, USA) equipped with an LWD 10x objective, analyzed, and quantified using the Columbus system (Perkin Elmer, Waltham, USA).

Induction of ROS

Brain glioma cells were seeded in 6-well plates at a density of 5×10^5 cells/well and were cultured for 24 h. Then, the culture media were replaced by fresh media containing free vinblastine, vinblastine liposomes, TfR-T_{12} modified vinblastine liposomes, stearyl-R_8 modified vinblastine liposomes or multifunctional vinblastine liposomes at a concentration of 30 nM vinblastine. After incubation for 6 h, the cells were collected and stained with DCFH-DA (ApplyGen, Beijing, China; 1 μM) at 37 °C for 0.5 h. Next, the cells were washed and re-suspended in PBS. The fluorescence intensity was measured by FCM. The excitation wavelength was set at 485 nm and the emission wavelength was set at 525 nm.

Apoptosis and autophagy mechanisms

Brain glioma cells were seeded in 96-well plates at a density of 5×10^3 cells/well and were cultured for 24 h. Then, drugs, including free vinblastine, vinblastine liposomes, TfR-T_{12} modified vinblastine liposomes, stearyl-R_8 modified vinblastine liposomes, and multifunctional vinblastine liposomes were added at a concentration of 30 nM vinblastine. After incubation for 6 h, the cells were fixed using 4% formaldehyde for 15 min and perforated using PBS containing 0.5% Triton X-100 and 0.3 M glycine for 15 min. Next, the cells were blocked using PBS (pH 7.4) containing 5% goat serum for 2 h. Afterwards, the cells were incubated with primary antibodies, including anti-caspase 3/7, anti-caspase 8, anti-caspase 9, anti-Bax, anti-cytochrome c, anti-Mcl-1, anti-FoxO1 and anti-LC3B antibodies (Beyotime, Beijing, China), at 4 °C overnight. Then, the cells were incubated with anti-rabbit or anti-mouse secondary antibody conjugated with Alexafluor-488 (ZSGB-BIO, Beijing, China) for 2 h. Finally, the cells were stained with Hoechst 33342 (5 μg/mL) at room temperature for 10 min. The fluorescence

intensity of each well was measured using the Operetta high content screening system and analyzed and quantified using the Columbus system.

Brain glioma-bearing mouse model

All of the animal experiments adhered to the principles of care and use of laboratory animals and were approved by the Institutional Animal Care and Use Committee of Peking University. Male BALB/c nude mice (Department of Laboratory Animal Science, Peking University, Beijing, China; 18-20 g) were anesthetized and fixed on a stereotaxic apparatus (RWD Life Science, Beijing, China). Next, an incision was made and the cranium was drilled 1 mm to the posterior and 2 mm to the right of the bregma. Brain glioma cells were re-suspended in blank MEM culture medium at a density of 2×10^5 cells/3 μL and were injected into the brain at a rate of 1 μL/min. Afterwards, the burr hole was covered with bone wax, and the incision was sutured.

After 12 days, the mice were sacrificed and the brains were obtained. The brains were made into frozen sections (4 μm), fixed using 4% paraformaldehyde solution and stained following the Nissl staining method. The images were obtained using a Caikon XDS-300C system (Caikon, Beijing, China).

In vivo imaging in brain glioma-bearing mice

DiR was used as the fluorescent probe to indicate the distribution of multifunctional liposomes in brain glioma-bearing mice. The mice were inoculated as above. After 12 days, the mice were randomly divided into 6 groups and administered with physiological saline, free DiR, TfR-T_{12} modified DiR liposomes, stearyl-R_8 modified DiR liposomes or multifunctional DiR liposomes via the tail vein. The mice were scanned at 1, 3, 6, 12, 24, and 48 h using the

IVIS Spectrum Pre-clinical In Vivo Imaging System (Perkin Elmer, Waltham, USA). Next, the mice were sacrificed, and the major organs, including the brains, hearts, livers, spleens, lungs and kidneys, were obtained and photographed to observe the fluorescence signals of varying formulations.

Kaplan-Meier survival curves

The mice were inoculated as above. After 12 days, the brain glioma-bearing mice were randomly divided into 6 groups (9 mice in each group) and administered 50 μg/kg of physiological saline, free vinblastine, vinblastine liposomes, TfR-T_{12} modified vinblastine liposomes, stearyl-R_8 modified vinblastine liposomes and multifunctional vinblastine liposomes via the tail vein. The treatments were applied every three days (three times in total). The mice were observed afterwards and the survival rates were calculated.

Efficacy of treating glioma stem cells and safety observations

The mice were inoculated and treated as above. After drug treatments, the mice from each group (three mice from each group) were sacrificed to isolate the brain, heart, liver, lung, spleen and kidneys. The brains were made into frozen sections (4 μm). The slices were perforated using 0.2% Triton X-100 solution for 0.5 h and blocked using PBS (pH 7.4) containing 10% goat serum for 5 h. Afterwards, the slices were incubated with anti-nestin antibody (Abcam, Cambridge, UK) at 4 °C overnight and then incubated with Alexafluor-488 conjugated anti-rabbit secondary antibody and Hoechst 33342 for 1 h. The images were obtained using a confocal laser scanning fluorescence microscope.

The hearts, livers, lungs, spleens and kidneys were fixed using 4% paraformaldehyde solution and then made into paraffin sections (4 μm). Afterwards, the slices were stained

following the hematoxylin-eosin (HE) staining method. The images were obtained using a Caikon XDS-300C system.

Statistical analysis

The data are represented as the means ± standard deviation. Analysis of variance (ANOVA) was used to determine the significance among groups, after which post hoc tests with the Bonferroni correction were used for multiple comparisons between individual groups. A value of $P < 0.05$ was considered to be significant.

Table S1. Characterization of the liposomes.

	Encapsulation efficiency (%)	Size (nm)	PDI	Zeta Potential (mV)
Vinblastine liposomes	99.0 ± 0.1	96.1 ± 1.2	0.176 ± 0.013	-7.47 ± 0.64
TfR-T_{12} modified vinblastine liposomes	97.0 ± 0.1	99.9 ± 2.2	0.225 ± 0.026	-6.68 ± 0.14
Stearyl-R_8 modified vinblastine liposomes	97.7 ± 0.1	115.4 ± 1.4	0.253 ± 0.008	10.37 ± 0.31
Multifunctional vinblastine liposomes	97.3 ± 0.2	112.4 ± 1.5	0.252 ± 0.007	-0.97 ± 0.17

Data are presented as mean ± standard deviation (n=3).

Table S2. Life span of brain glioma-bearing mice after treatment with varying formulations.

Groups	Survival time (days)			Increased life span% (ILS%)	
	Survival range	Mean ± SD	Median	Mean	Median
Physiological saline	14-22	18.50 ± 1.02	19	—	—
Free vinblastine	15-25	22.00 ± 0.97	20	5.81%	5.26%
Vinblastine liposomes	14-26	24.33 ± 1.05	21	7.56%	10.53%
Tfr-T_{12} modified vinblastine liposomes	22-31	26.50 ± 1.02	26	37.21%	36.84%
Stearyl-R_8 modified vinblastine liposomes	22-32	29.17 ± 4.17	27	41.86%	42.11%
Multifunctional vinblastine liposomes	23-33	29.83 ± 5.56	29	45.93%	52.63%

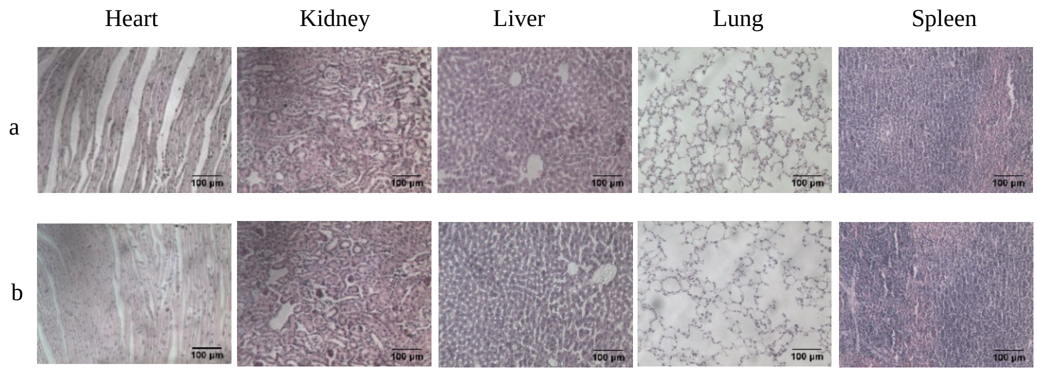

Figure S1. Safety evaluation of multifunctional vinblastine liposomes.

Note: Hematoxylin-eosin staining of organs from glioma-bearing mice: a, after treatment with physiological saline; b, after treatment with multifunctional vinblastine liposomes.
